# Low frequency of NS5A relevant resistance-associated substitutions to Elbasvir among hepatitis C virus genotype 1a in Spain: a cross-sectional study

**DOI:** 10.1038/s41598-017-02968-7

**Published:** 2017-06-06

**Authors:** Claudia Palladino, Marta Sánchez-Carrillo, Irene Mate-Cano, Sonia Vázquez-Morón, Ma Ángeles Jimenez-Sousa, Mónica Gutiérrez-Rivas, Salvador Resino, Verónica Briz

**Affiliations:** 1 Research Institute for Medicines (iMed.ULisboa), Faculty of Pharmacy, University of Lisbon, Lisbon, Portugal; 2 Laboratory of Viral Hepatitis, National Center for Microbiology, Institute of Health Carlos III, Majadahonda Madrid, Spain; 3 Infectious Disease Department, Henares Hospital, Madrid, Spain

## Abstract

Relevant resistance-associated substitutions (RASs) to elbasvir, the new HCV NS5A inhibitor, may limit its efficacy and lead to virological failure in HCV-GT1a-infected patients. There are few data outside clinical trials evaluating their prevalence and impact of elbasvir/grazoprevir. A multicenter cross-sectional study of 617 HCV-GT1a-infected individuals attended in 84 Spanish hospitals from the 17 Autonomous Communities and two Autonomous cities was performed. HCV population sequencing was used to identify RASs to elbasvir and the mutational pattern and drug sensitivity were confirmed by geno2pheno_[HCV]_. Viruses bearing RASs to elbasvir were present in 6.2% of HCV-GT1a infected patients. The most common RASs were the Y93C/H/N and Q30E/H/R (2.4% and 2.3%; respectively). Only 3.4% of patients had viruses with RASs that confer reduced susceptibility to elbasvir by geno2pheno_[HCV]_ that identified exclusively the positions Q30H/R (n = 7) and Y93C/H/N (n = 8) as single mutations and Q30H + Y93H (n = 4) and Q30R + Y93H (n = 2) as double mutations considered as RASs to elbasvir. Lower prevalence of RASs to elbasvir in our HCV-GT1a-Spanish cohort was observed than reported previously in clinical trials. This information may be essential to guiding the implementation of elbasvir/grazoprevir in Spain, expected at the beginning of 2017 and the management of GT1a-infected patients.

## Introduction

Elbasvir/grazoprevir has been approved by the Food and Drug Administration in January 2016^1^. In the European Union, it was approved in July 2016 and included on the list of additionally monitored medicines by the European Medicines Agency^2^. Elbasvir/grazoprevir represents a new combination product of direct-acting antivirals (DAAs) recently incorporated into the therapeutic armamentarium to treat the hepatitis C virus (HCV). Grazoprevir belongs to the HCV NS3/4A protease inhibitor (PI) family and elbasvir represents one of the newest HCV NS5A inhibitors.

Elbasvir/grazoprevir combination therapy showed a high efficacy in treatment-naïve patients with chronic HCV enrolled in the phase 3 C-EDGE randomized, placebo-controlled trial^3, 4^. 95% of subjects achieved a sustained virological response at 12 weeks (SVR12), with an efficacy of 92% for genotype 1a, 99% for genotype 1b, 100% for genotype 4 and 80% for genotype 6. However, the trial included relatively few individuals with genotype 4 and 6 infections.

The presence of resistance-associated substitutions (RASs) may reduce HCV sensitivity to this new DAA, thus limiting its efficacy. Previous studies have shown that the presence of RASs in the NS3 region does not affect the efficacy of grazoprevir in PI-naïve patients or those who have been exposed mainly to early PIs like boceprevir and telaprevir, which may not select for the same NS3 substitutions as more advanced PIs^3^.

However, RASs in the NS5A region have an impact on the efficacy of elbasvir. The impact of pre-existing HCV variants on treatment response in naïve HCV-infected subjects treated with elbasvir/grazoprevir has been assessed in the C-EDGE TN study. NS5A RASs to elbasvir were identified in 12% of participants (specifically from the United States) harboring genotype 1a (GT1a) and were associated with a reduction in the sustained virological response at 12 weeks (SVR12) to 58%^3^. The recommendation to extend the duration of elbasvir/grazoprevir treatment to 16 weeks with inclusion of ribavirin for HCV-GT1a treatment-naïve patients with NS5A RASs is based on the results of the C-EDGE TE^5^. Specifically, baseline NS5A variants with >5-fold shift to elbasvir reduced SVR12 to 52% in HCV-GT1a-infected subjects, while the SVR12 increased to 95% in the intention-to-treat analysis when ribavirin was added to elbasvir/grazoprevir and the treatment was extended to 16 weeks.

Therapy failure with elbasvir in HCV-GT1a-infected patients has been associated with the presence of the specific substitutions M28A/G/T, Q30D/E/H/G/K/L/R, L31F/M/V, H58D and Y93C/H/N/S, which are a subset of the NS5A class RASs^6^. Notably, the same substitutions have been considered as clinically relevant RASs at NS5A specific to elbasvir in the latest European guidelines released in September 2016^7^ and American guidelines^8^.

Cross-resistance between NS5A RASs to elbasvir and the approved first-generation NS5A inhibitors have been documented^6, 9^, as well as the highly persistent nature of these substitutions in the viral population once established^10, 11^. Both phenomena may limit the use of the NS5A inhibitor family. As a consequence, NS5A resistance testing is recommended for HCV-GT1a-infected patients who are eligible for treatment with elbasvir/grazoprevir^8^. NS5A polymorphisms seem to have a modest influence on the efficacy of elbasvir/grazoprevir combination for HCV genotype 1b. Previous studies have shown that treatment-naïve GT1b-patients that harbored viruses with RASs of 94% achieved SVR12. Moreover, no impact on HCV genotype 4 has been observed and a higher impact on GT6 (SVR12 of 67%) has been also reported^3^.

Within genotype 1a, two divergent clades (I and II) have been described^12^. Interestingly, recent studies have highlighted distinct spatial distribution of these two clades, with clade I being more represented in the United States and both clades equally distributed in Europe^13, 14^, as well as different associations with the presence of naturally occurring resistance mutations to the NS3 protease inhibitors^13^.

In Spain, elbasvir/grazoprevir is scheduled to come into clinical use at the beginning of 2017. HCV GT1a represents 23% of all HCV infections in Spain, while GT1b represents 31%^15^. Therefore, previous knowledge of the presence of NS5A RASs to EBR in the Spanish population infected with HCV-GT1a would help to identify the proportion of patients who may benefit from this new treatment. In the present study, a RASs analysis using Sanger sequencing detection was performed in HCV-GT1a-infected patients from Spain to evaluate the prevalence and impact of clinically relevant NS5A RASs to the new NS5A inhibitor.

## Results

Of 632 patients included in this study, 13 samples (2.1%) were excluded because HCV RNA could not be amplified or we were unable to achieve a HCV sequence consensus by Sanger sequencing. In total, 617 patients with HCV-GT1a were available for statistical analysis. Overall, 80.1% (n = 494) of subjects were men and had a median age of 50 years (47–53). Furthermore, 52.8% (n = 326) were HCV-monoinfected patients and 47.2% (n = 291) were HIV/HCV-coinfected patients. No differences between clades were observed when determined in the NS5A or NS3 genes. HCV-GT1a clade II was more prevalent than clade I (82.5%, n = 509, vs 17.5%, n = 108), as were HCV-monoinfected patients in comparison to HIV/HCV-coinfected individuals (87.4% vs 77.0%, respectively, (*P* = 0.001) (Supplementary File 1).

**Figure 1:**
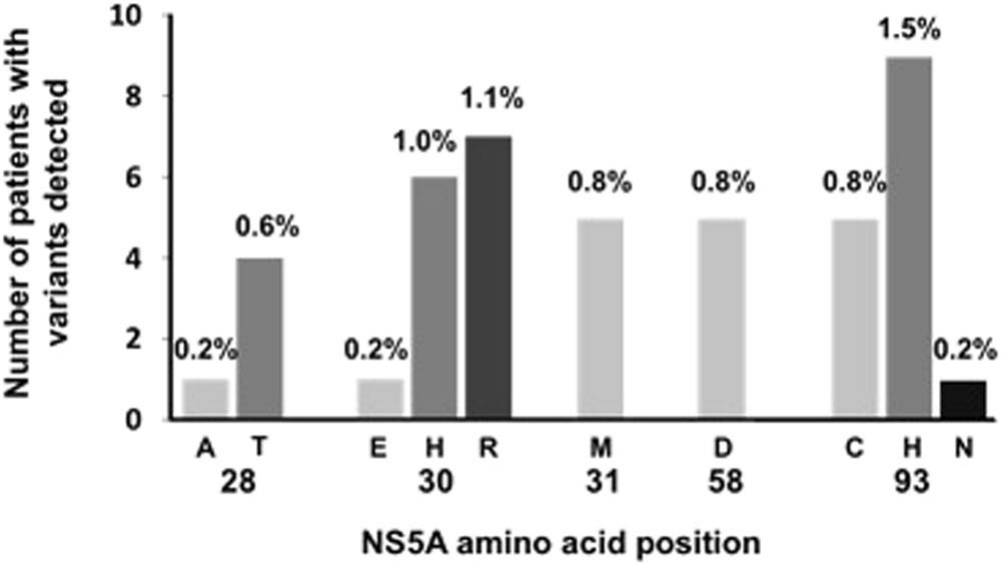
Types and prevalence of NS5A RASs to elbasvir detected in HCV GT1a-infected patients never treated with any NS5A inhibitor family in Spanish.

Viruses bearing RASs to elbasvir were present in 38 samples (6.2%) of HCV-GT1a-infected patients (Fig. 1), six of those having viruses with double RASs. The most common RASs were Y93C/H/N (2.4%; n = 15) and Q30E/H/R (2.3%; n = 14), while M28A/T, L31M and H58D were less represented (0.8%, n = 5, each). The double mutations Q30H + Y93H and Q30R + Y93H had a low frequency (0.6%, n = 4, and 0.3%, n = 2) (Fig. 1). Interestingly, 3.4% (n = 21) of patients had viruses with RASs that confer reduced susceptibility to elbasvir and were identified according to g2p_[HCV]_. This algorithm exclusively identified the positions Q30H/R (n = 7) and Y93C/H/N (n = 8) as single mutations and Q30H + Y93H (n = 4) and Q30R + Y93H (n = 2) as double mutations considered as RASs to elbasvir.

**Figure 2:**
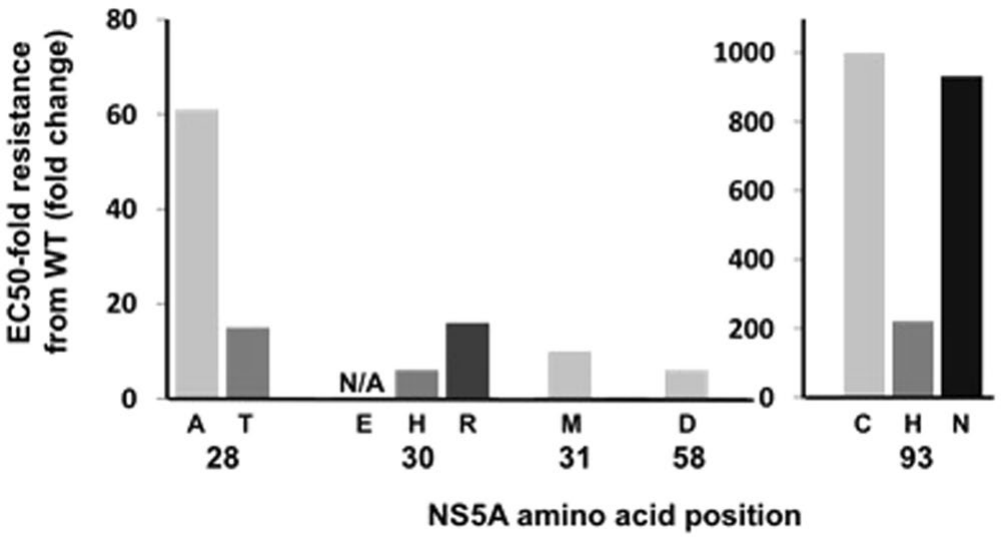
Impact of the NS5A substitution variants according to the fold change. Abbreviations: WT, wild type; EC50, concentration required to achieve 50% inhibition of HCV replication; NS5A, nonstructural protein 5 A.

Subsequently, the impact of the identified variants according to the fold-change NS5A RASs to elbasvir was evaluated in order to assess the specific weight of each mutation and thus the phenotypic impact of the variants found in our cohort (Fig. 2). The mutations identified in the analyzed sequences presented a different pattern of resistance to elbasvir, ranging from 6x to 1235x (Fig. 2)^10^. In particular, we identified mutations in positions Q30H/R and Y93C/H/N as having the highest-level resistance to elbasvir (Fig. 2). Next, it was also assessed whether the different clades may influence the presence of RASs to elbasvir, but no differences were observed between clades (clade I: 8.3%, n = 9/108, vs clade II: 5.7%, n = 29/509; (*P* = 0.278). Remarkably, the patients harboring viruses with NS5A RASs that conferred reduced susceptibility to elbasvir were twice as prevalent among HIV/HCV-coinfected patients than HCV-monoinfected patients (8.2%, n = 24/291, vs 4.3%, n = 14/326; *P* = 0.045).

Furthermore, the prevalence of the M28V polymorphism was 2.9 (18/617), observed in 2.4% (n = 7/291) of HIV/HCV-coinfected and 3.4% (n = 11/326) of HCV-monoinfected patients, with 2/11 of the HCV-monoinfected also having another NS5A RAS (30R).

Six individuals had the double mutations Q30H + Y93H and Q30R + Y93H. Fold change analysis was based on Black *et al*. 2015^10^ and Cento *et al*. 2015^16^.

## Discussion

In this study, we found a low prevalence of NS5A RASs to elbasvir in HCV-GT1a-infected patients never treated with any NS5A inhibitor. To our knowledge, this is the first Spanish nationwide survey of the prevalence of NS5A RASs in HCV-GT1a. Our data of prevalence of NS5A RASs to elbasvir (6.2%) contrasts with the prevalence of 12% (19/154) recently reported by Zeuzem *et al*. (2015) in treatment-naïve HCV-GT1a-infected patients enrolled in the phase 3 C-EDGE TN trial^3, 4^. Interestingly, when the same polymorphisms were compared between the C-EDGE TN trial and our study (with the M28V polymorphism included) a lower prevalence in the RAS analyzed in our study population was still present (8,8% vs 12%). The number of HCV-GT1a patients enrolled in our cohort and in the C-EDGE TN trial differs (617 vs 154), which may have an influence on the differences observed. In addition, the lack of data regarding HCV viral load in our cohort prevents us from assessing the differences in elbasvir NS5A RAS prevalence according to higher (>800.000 UI/mL) or lower (≤800.000 UI/mL) HCV viral loads, which may also influence the results.

The effect of NS5A RASs to elbasvir in response to HCV treatment in real-world settings is still unclear and their characterization and impact on first-line HCV therapy and retreatment strategies remains a great challenge. Moreover, the lack of data for NS5A RASs to elbasvir in the clinical practice prevents us from comparing our data with those from different countries.

Susser *et al*. (EASL 2016) reported a prevalence of 13% of resistance-associated variants to NS5A in a real world setting focusing on daclatasvir, ledipasvir and ombitasvir NS5A inhibitors^17^. According to Jacobson *et al*. (2015) elbasvir has been associated with treatment failure in HCV-GT1a-infected patients in the presence of the specific substitutions M28A/G/T, Q30D/E/H/G/K/L/R, L31F/M/V, H58D and Y93C/H/N/S, which are a subset of the NS5A class RASs^6^. The evaluation of a specific subset of the NS5A class RAS in our cohort that may confer resistance to elbasvir and not all NS5A inhibitors, has surely influenced the lower prevalence observed in our cohort when compared with Susser *et al*. (EASL 2016) data. For instance, the presence of any RAS in the positions 24, 32, 38 and 92 that have been included in Susser and colleagues (EASL 2016) were not included in our study because no association with elbasvir failure has been observed to date.

Although the resistance patterns observed *in vitro* and *in vivo* may do not necessarily correlate, Nakamoto and colleagues (2014) reported a correlation between resistant variants emerging *in vivo* to the NS5A inhibitors daclatasvir and ledipasvir and resistant variants detected in the *in vitro* HCV replicon system^18^. However, a higher complexity of linked variant combinations has been observed in clinical samples from patients treated with daclatasvir in 14-day monotherapy when compared *in vitro* and *in vivo* resistance patterns^19^ that may be explained by the higher heterogeneity of HCV sequences derived from clinical specimens compared to replicons^20^.

Recent studies have unveiled distinct spatial distributions of the two HCV-GT1a clades, with both clades equally distributed in Europe and clade I being more prevalent in the United States^13, 14^. In our study, we found a much higher prevalence of clade II than clade I, the former representing approximately 4 of 5 sequences analyzed and the latter significantly less prevalent (17%) than in other European countries (48% for Italian sequences, 53% for German). This phylogenetic difference may indicate a difference in temporal spread of the HCV-GT1a clades in Spain as compared to other European countries, where both clades are equally distributed. In addition, we observed an inverse association of clade II with the presence of HIV coinfection, in contrast with data reported by de Luca *et al*.^13^. Differences in the prevalence of NS5A RASs to elbasvir according to HCV-GT1a clades may provide some explanation for those variants being less frequent in HCV-GT1a-infected patients from Europe than North America, where clade I is more prevalent^13^. In our study population, the low prevalence of NS5A RASs to elbasvir and of HCV-GT1a clade I did not allow for the establishment of any association between the HCV-GT1a clade and the presence of NS5A RASs. Therefore, further studies with larger cohorts are warranted to clarify the emergence of NS5A RASs to elbasvir according to the HCV-GT1a clade.

Resistance tests to identify RASs in HCV-GT1a infected patients eligible for elbasvir/grazoprevir initiation are recommended^11^. In fact, the presence of these NS5A RASs and their effect in HCV treatment response will influence treatment selection in the future. Pretreatment resistance analyses can optimize treatment selection although, according to the low prevalence identified in our cohort, today perhaps it would not be necessary to perform tests of NS5A RASs routinely in Spain. However, this scenario is likely to change and therefore it would be necessary to implement a monitoring system that would allow us to evaluate the necessity for routinely carried out NS5A RASs tests in the near future.

It is important to mention that elbasvir/grazoprevir may be the most cost-effective treatment compared to the current DAAs approved, which is likely to influence treatment choice^21^. This and the low prevalence of NS5A RASs to elbasvir observed in our cohort will most certainly encourage the implementation of elbasvir/grazoprevir as the first-line option in Spain.

Several limitations have to be taken into account. Firstly, the lack of clinical data did not permit us to assess the possible impact of NS5A RASs to elbasvir on the virological response. Nevertheless, the clinically relevant RASs to elbasvir within NS5A were extrapolated from the recently reviewed European Guidelines^7^. Secondly, epidemiological and clinical data (e.g., HCV RNA viral load, HCV transmission mode) were limited and would be required for a comprehensive interpretation of the results. For instance, despite the fact that the most recent strategic plan for tackling HCV in the Spanish National Health System set out the general criteria for DAAs treatment, indicating as top priority those patients with advanced liver fibrosis (F2–F4) and patients included in a waiting list for transplant or those already transplanted^22^, we cannot guarantee that all patients enrolled in this study presented stages of advanced liver fibrosis. In spite of this, it is worth mentioning that our study uses a nationwide representative sample of the population living with chronic HCV infection in Spain. That may help us to form a general picture of the actual situation for the entire Spanish population, unlike studies relating to individual hospitals. Thirdly, Sanger sequencing (HCV population sequencing) was used to identify NS5A RASs instead of deep sequencing. Although the use of population sequencing may slightly underestimate the prevalence of NS5A RASs to elbasvir, their impact on efficacy to elbasvir/grazoprevir seems to disappear when given 16 weeks of elbasvir/grazoprevir + RBV^6^. Besides, the need for a rapid diagnosis of NS5A RASs may rule out/discourage deep sequencing as an option due to higher turnaround time and cost.

In conclusion, our data show that naïve HCV-GT1a-infected patients had a low prevalence of NS5A RASs to elbasvir in Spain. This information may be essential to guiding the implementation of this DAA in Spain and the management of HCV-GT1a-infected patients in the near future.

## Methods

### Study Design and Patients

A multicenter cross-sectional study of 632 chronically-infected individuals with HCV-GT1a and naïve to NS5A inhibitors was carried out. The STROBE checklist was used to help design and conduct the study^23^.

The patients were attended in 84 Spanish health centers distributed throughout the national territory (Appendix, Supplementary File 2). The samples were collected prior to anti-NS5A HCV therapy initiation, between October 2014 and February 2015. Genotyping testing, in order to correctly identify subtype GT1a was done by the commercial assay Real-Time HCV genotype II (Abbott), following the manufacturer’s instructions^24^.

The plasma specimens were sent to the National Center of Microbiology (*Instituto de Salud Carlos III* [ISCIII]) for the determination of NS5A RASs together with a minimum data set (patient code, age, gender, HIV infection, hospital, and region). Both data and samples were anonymized and transferred to the ISCIII National Biobank (Ref.: B.0000984).

### Ethics

The study was conducted in accordance with the Declaration of Helsinki. The Institutional Review Board and the Research Ethic Committee of ISCIII approved the study (No. CEI PI 43_2015).

### Resistance-Associated Substitutions

Elbasvir-specific clinically relevant RASs classified as variants at NS5A specific substitution were: M28A/G/T, Q30D/E/H/G/K/L/R, L31F/M/V, H58D and Y93C/H/N/S according to the latest European guidelines^7^. The presence of the M28V polymorphism known to be possibly involved in resistance to elbasvir was also assessed.

### Amplification and sequencing of HCV NS5A

Viral RNA was extracted from plasma with the QIAsymphony DSP Virus/Pathogen Kit (Qiagen, Hilden, Germany) and complete NS5A gene amplification was performed using the RT-PCR OneStep kit (Qiagen, Hilden, Germany) using the oligonucleotides NS4BFW (5′TGAGGCGACTVCACCAGTGG3′) and NS5BRV (5′TCTTCCGCGGCRCACGGGGTGA3′). Amplification was programmed as follows: 30 min at 54 °C; 15 min at 95 °C; 35 repetitive cycles of 30 sec at 94 °C, 30 sec at 60 °C and 2 min at 72 °C in the Applied BiosystemsVerityTM Thermal Cycler. Negative and positive controls were included in all amplification procedures. Positive PCR products were visualized with GelRed (Biotium USA) at a HCV specific band size of ~1,343 bp. Amplicons were purified (illustraTMGFXTM PCR DNA and Gel Band Purification Kit, GE Healthcare, USA) and diluted 1:2 using nuclease free water (Roche). Subsequently, the sequencing reaction was performed with the following oligonucleotides: FwSc2 (5′CGACTRCACCAGTGGATAAGC3′); FwSc3 (5′CTRCACCAGTGGATAAGCTCG3′); FwSc5 (5′CCCATTAACGCCTACACCACG3′); FwSc7 (5′CCTGACGCCGAGCTCATAGAG3′); RvSc3 (5′AGCGAGTGTGCATGATGCCAT3′); RvSc7 (5′GTGCGCCTGTCCAGGAATAAGA3′) and Sanger sequencing was performed (ABI PRISM 377 DNA sequencer, Applied Biosystems, Foster City, CA) (with a sensitivity threshold of approximately 15%)^25^.

### Bioinformatic analysis

SeqMan program (Lasergene DNASTAR Inc, Madison, WI, USA) was used to get the consensus sequences and MEGA6 program (MEGA6: Molecular Evolutionary Genetics Analysis Version 6.0; http://www.megasoftware.net/) was used to align the NS5A sequences with the H77 representative HCV-1a sequence. The NS5A gene was analyzed to determine the prevalence of NS5A RASs to EBR in patients that never received any NS5A inhibitor and HCV-GT1a according to the recent European guidelines^7^. The NS5A mutational pattern and drug sensitivity were confirmed by geno2pheno [HCV] (g2p_[HCV]_) (Bonn, Germany; http://hcv.geno2pheno.org/). The HCV-GT1a lineages (clade I and clade II) were identified with g2p_[HCV]_ using both NS5A and NS3 regions. The isolate H77 (GenBank accession number AF009606) was used as GT1a reference sequence. The level of resistance to EBR (fold-change) of each RAV identified in our cohort was assessed according to Black *et al*. 2015^10^.

### Statistical analysis

Categorical variables were analyzed using the chi-squared test or Fisher’s exact test and continuous variables were compared by Mann-Whitney U test. *P*-values were 2-tailed and statistical significance was defined as *P* < 0.05. Statistical analyses were performed using the SPSS software Version 22.0 (IBM Corp, Chicago, Armonk, NY, USA).

## Electronic supplementary material

Supplementary Information
