# Supplementary material for: Low frequency of NS5A relevant resistance-associated substitutions to Elbasvir among hepatitis C virus genotype 1a in Spain: a cross-sectional study
Source: Sci Rep. 2017 Jun 6;7:2892. doi: 10.1038/s41598-017-02968-7 (PMC5460287; doi:10.1038/s41598-017-02968-7)
Supplement: Supplementary file 1 — Supplementary Information [file 41598_2017_2968_MOESM1_ESM.doc]

**Low frequency of NS5A relevant resistance-associated substitutions to Elbasvir among hepatitis C virus genotype 1a in**

**Spain: a cross-sectional study**

**Authors**: Claudia Palladino1, Marta Sánchez-Carrillo2, Irene Mate-Cano3, Sonia

Vázquez-Morón2, Mª Ángeles Jimenez-Sousa2, Mónica Gutiérrez-Rivas2, Salvador

Resino2+, Verónica Briz2+* on behalf of the Spanish Group of Chronic Viral Hepatitis

1. Research Institute for Medicines (iMed.ULisboa), Faculty of Pharmacy, University of

Lisbon, Lisbon, Portugal.

2. Laboratory of Viral Hepatitis, National Center for Microbiology, Institute of Health

Carlos III, Majadahonda. Madrid, Spain.

3. Infectious Disease Department. Henares Hospital. Madrid, Spain.

**Supplementary file 1**. Epidemiological characteristics of patients included in the study.

| Characteristics | All patients | HIV status | | *P* |
| --- | --- | --- | --- | --- |
|  |  | **HCV-monoinfected** | **HIV/HCV-coinfected** |  |
| **No.** (%) | 617 | 326 (52.8) | 291 (47.2%) |  |
| **Age, years** (median, IQR) | 50 (47; 53) | 51 (47; 54) | 49 (46; 53) | 0.001* |
| **Sex** (n, %) |  |  |  |  |
| Men | 494 (80.1) | 248 (76.1) | 246 (84.5) | 0.009** |
| Women | 123 (19.0) | 78 (23.9) | 45 (15.5) |  |
| **HCV clade** (n, %) |  |  |  |  |
| I | 108 (17.5) | 41 (12.6) | 67 (23.0) | 0.001** |
| II | 509 (82.5) | 285 (87.4) | 224 (77.0) |  |

Notes: IQR, interquartile range; * Mann-Whitney test; ** Pearson Chi-Square (all the tests are 2-sided).

**Supplementary file 2.** **Appendix**

**Centers and investigators involved in Spanish Group of Chronic Viral Hepatitis participating in this epidemiological survey**

1. Área Sanitaria De Ferrol (A Coruña): Patricia Ordoñez Barrosa.

2. Centro Médico De Asturias (Asturias): Rosario Vilches Vilches.

1. Complejo Asistencial Universitario De Burgos (Burgos): Federico Saez-Royuela Gonzalo.
2. Complejo Hospitalario De Navarra (Navarra): Aitziber Aguinaga Pérez, Isabel Polo Vigas.
3. Complexo Hospitalario Universitario De Ourense (Ourense): Gloria Esteban Meruéndano.
4. Complexo Hospitalario De Pontevedra (Pontevedra): Patricia Álvarez García, Trigo Daporta Matilde.
5. Complexo Hospitalario Universitario De Santiago (A Coruña), Antonio Aguilera Guirao.
6. Complexo Hospitalario Universitario A Coruña (A Coruña): María Ángeles Cañizares Castellanos.
7. Complexo Hospitalario Xeral-Calde (Lugo): Amparo Coira Nieto.
8. Fundación Hospital Alcorcón (Madrid): María Luisa Casas Losada.
9. Fundación Hospital De Jove (Asturias): Elisa Hidalgo Pérez.
10. Fundación Jiménez Díaz-Ute (Madrid): Ricardo Fernández Roblas.
11. Gerencia De Área De Salud De Badajoz-Llerena-Zafra (Badajoz): Gracia Sanchez Alor.
12. Gestión Sanitaria De Mallorca (Mallorca): Victoria Fernández Baca Gutiérrez Del Álamo.
13. Hospital Arnau De Vilanova (Valencia): Remedios Giner Duran.
14. Hospital Can Misses (Ibiza): Adoración Hurtado Fernández.
15. Hospital Central De Asturias (Asturias): Santiago Melón García.
16. Hospital Central De La Defensa Gómez-Ulla (Madrid): María Mateo Maestre.
17. Hospital Clinic (Barcelona): Francesc Marco Reverte.
18. Hospital Clínico San Carlos (Madrid): Esther Culebras Lopez, Icíar Rodríguez Avial, Jorge Vergas García.
19. Hospital Clínico Universitario de Salamanca (Salamanca): María Nieves Gutierrez Zufiaurre.
20. Hospital Clínico Universitario de Valladolid (Valladolid): Carmen Hinojosa Mena Bernal, Sara Lorenzo Pelayo.
21. Hospital Clínico Universitario Lozano Blesa (Zaragoza): Rafael Benito Ruesca, Carmen Rubio Calvo.
22. Hospital Comarcal De Inca (Mallorca): Juan Saurina Gomila.
23. Hospital De Basurto (Vizkaya): Silvia Hernaez Crespo, Paloma Liendo Arenzana, María Dolores Suárez Fernández.
24. Hospital De Cabueñes (Asturias): Asunción Del Valle Prieto, Luis Otero Guerra.
25. Hospital De Cruces (Vizcaya): Luis Elorduy Otzua, Patricia Iraurgui Aracarazo, Leire López Soria.
26. Hospital De Donostia (Guipúzcoa):Carlos Gustavo Cilla Eguiluz.
27. Hospital De Especialidades De Jerez De La Frontera (Cádiz): María Dolores López Prieto Jose, Luis de Francisco Ramírez.
28. Hospital De Especialidades De Puerto Real (Cádiz): Iria Jesús De La Calle.
29. Hospital De Gran Canaria Dr. Negrín (Las Palmas): María José Pena López.
30. Hospital De Hellín (Albacete): Carmen Romero Portilla.
31. Hospital De La Agencia Valenciana De Salud Vega Baja (Alicante) Nieves Gonzalo Jiménez.
32. Hospital De La Línea De La Concepción (Cádiz): Francisco Javier Casas Ciria.
33. Hospital De Móstoles (Madrid): Fátima López Fabal.
34. Hospital De Palamós (Girona): Nuria Guinart Sola.
35. Hospital De Poniente (Almería): Teresa Cabezas Fernandez.
36. Hospital Do Meixoeiro (Pontevedra): Sonia Pérez Castro.
37. Hospital Don Benito-Villanueva de La Serena (Badajoz): Saray Rodríguez Garrido, Antonio Valle Valencia.
38. Hospital El Bierzo (León): Carmen Raya Fernández.
39. Hospital Ernest Lluch Martin (Zaragoza): Blanca Fortuño Cebamanos.
40. Hospital Galdakao-Usansolo (Vizcaya): María José López De Goicoechea San Román.
41. Hospital Garcia Orcoyen (Navarra): Laura Barrado Blanco.
42. Hospital General de Castellón (Castellón): Bárbara Gomila Sard, Susana Sabater Vidal.
43. Hospital General De Lanzarote (Las Palmas): Rodolfo Copado Carretero.
44. Hospital General De Segovia (Segovia): Jorge Elizaga Corrales.
45. Hospital General Juan Ramón Jiménez (Huelva): José Saavedra Martin.
46. Hospital General Río Carrión (Palencia): Teresa García Valero.
47. Hospital General San Jorge (Huesca): Luis Torres Sopena.
48. Hospital General Universitario De Alicante (Alicante): Adelina Gimeno Gascón.
49. Hospital General Universitario De Elche (Alicante): Monserrat Ruiz García.
50. Hospital General Universitario Gregorio Marañón (Madrid): Teresa Vicente Rangel.
51. Hospital General Universitario Reina Sofía (Murcia): Antonio Jesús Marín Cervantes.
52. Hospital Infanta Cristina (Badajoz): Rosa Sánchez Silos.
53. Hospital Infanta Elena-Complejo Hospitalario Universitario de Huelva (Huelva): Matilde De La Iglesia Salgado.
54. Hospital J.M. Morales Meseguer (Murcia): Carmen Guerrero Gómez.
55. Hospital General Mateu Orfila (Menorca): Luis Carbo.
56. Hospital Nuestra Señora De Sonsoles (Ávila): Antonio Gómez Del Campo Dechado.
57. Hospital Povisa S.A (Pontevedra): Raquel Baluja Pino, María Dolores Martinez Otero.
58. Hospital Puerta De Hierro (Majadahonda): Francisca Portero Azorin.
59. Hospital General Universitario Rafael Méndez de Lorca (Lorca): Eva Cascales Alcolea, Jose María Zarauz García.
60. Hospital Universitario Reina Sofia (Córdoba): Fernando Rodríguez Cantalejo.
61. Hospital Reina Sofia (Tudela): Jose Javier García Irure.
62. Hospital Universitario San Agustín (Asturias): Gema Sierra Dorado.
63. Hospital San Pedro (Logroño): Consuelo Martinez Gil, María Victoria Muruzabal Sitges.
64. Hospital Santa Maria Nai (Ourense): Juan Garcia Costa.
65. Hospital Universitario Araba-Txagorritxu (Álava): María Jesús Lezaun Bugui.
66. Hospital Universitario Marques De Valdecilla (Santander): Javier Crespo García, Ana Saez López.
67. Hospital Universitario De Canarias (Tenerife): Felicitas Díaz-Flores Estevez.
68. Hospital Universitario De Ceuta (Ceuta): Jose López Barba.
69. Hospital Universitario De La Princesa (Madrid): Laura Cardeñoso Domingo.
70. Hospital Universitario Doctor Peset (Valencia): Juan Alberola Enguidanos.
71. Hospital Universitario Insular De Gran Canaria (Las Palmas): Evora Santana Rodriguez.
72. Hospital Universitario Miguel Servet (Zaragoza): Ana Martínez Sapiña.
73. Hospital Vital Álvarez Buylla (Asturias): María Carmen Galarraga Gay.
74. Hospital Virgen De La Concha (Zamora): Rosa Martínez González.
75. Hospital Virgen De La Salud (Toledo): César Gómez Hernando, Pilar Zamarrón Fuertes.
76. Hospital Virgen De La Victoria (Málaga): Encarnación Clavijo Frutos.
77. Hospital Virgen Del Castillo (Murcia): María Luisa López Yepes.
78. Hospital Virgen Del Puerto (Plasencia): Carlos García Tejero, José Román Muñoz del Rey.
79. Hospital Universitario Virgen Del Rocío (Sevilla): Laura Merino Díaz.
80. Laboratorio De Referencia Del Camp De Tarragona i Terres De L´Ebre (Tarragona): María Jose Puerta Martínez.
81. Laboratorio Referencia Catalunya (El Prat De Llobregat): Margarita Salvado Costa, Gloria Soria Guerrero.
82. Laboratorio Br salud Ut (Madrid): Esteban Aznar Cano.
